# Some Pathological Data on 2000 Adenocarcinomas and Squamous Cell Carcinomas of the Lung

**DOI:** 10.1038/bjc.1963.1

**Published:** 1963-03

**Authors:** W. I. B. Onuigbo


### BRITISH JOURNAL OF CANCER

VOL. XVII | MARCH, 1963 | NO. 1

### SOME PATHOLOGICAL DATA ON 2000 ADENOCARCINOMAS AND SQUAMOUS CELL CARCINOMAS OF THE LUNG

W. I. B. ONUIGBO

*From the Department of Pathology, The University and Western Infirmary, Glasgow**

Received for publication November 26, 1962

A SALIENT feature of the published statistical analyses of lung cancer is the diversity of the answers given to several questions. One area of conflict is the influence of the cell type on the pathology of this tumour. Thus, according to Berkson (1953), who studied 76 cases, " It would seem fairly certain that the various types of (lung) cancer behave differently ", but, in the opinion of Budinger (1958), whose study covered 250 cases, " Certainly there was no apparent relation between histological type and pattern of spread ".

A survey of the literature suggests that the small size of most series is a major cause of the variability of the results. In addition, there are difficulties attributable to the histological pleomorphism of carcinomas of bronchial origin. Furthermore, as Nohl (1962) pointed out, authors' classifications of these tumours vary from as few as one to as many as seven types. However, most workers recognise at least two distinct types, namely, squamous cell carcinoma and adenocarcinoma. It is, therefore, with these two cell types alone that I propose to deal, the series of 2000 cases being large enough to reveal findings which are likely to be of fundamental importance.

### INVESTIGATION

7125 cases diagnosed by pathologists as primary carcinomas of the lung were collected during a country-wide survey of the necropsy records of 27 medical schools and 7 associated hospitals. After rejecting cases without histological reports and those of mixed or other cell types, there remained 2000 cases which were specifically reported as either squamous-celled (1299 cases) or adenocarcinomatous (701 cases). The bulk of these cases were recorded in recent years, mostly in the 1950s and late forties ; only 57 cases dated back to the 1930s. I have not personally verified the histological material, but have carried out a detailed comparison of the two recorded cell types, with special reference to sex, age, frequency of extrathoracic metastases, organ selectivity and laterality of the metastases of paired organs.

* Present address : Department of Pathology, Lagos University Medical School, Lagos, Nigeria.

### RESULTS AND COMMENTS

### *Sex*

Table I shows the sex distribution of the whole series according to the different hospitals. Of the 2000 cases analysed, there were 1716 men and 284 women,

TABLE I.—*The Distribution of the Cell Types according to Sex*

| Cell type | Men | Women | Totals |
|---|---|---|---|
| Squamous cell Carcinoma | 1182 | 117 | 1299 |
| Adenocarcinoma | 534 | 167 | 701 |
| Totals | 1716 | 284 | 2000 |

i.e., a ratio of 6·04 : 1. This compares with the ratio of 6·05 : 1 reported by Strauss and Weller (1957) in an analysis of 296 cases of all histological types of lung cancer necropsied at the University of Michigan Medical School. As these authors remarked, their own ratio was well within the range of general experience in the United States. The results of an independent survey (Galluzi and Payne, 1955) of 741 cases collected from British hospitals do not differ widely. Therefore, the present series is in all probability a representative one.

Among the 1716 men (Table I), 1182 exhibited squamous cell carcinoma, whereas 534 displayed adenocarcinomatous tumours—a ratio of 2·21 : 1. Clearly men are less prone to develop adenocarcinoma than squamous cell carcinoma. Nevertheless, it is noteworthy that this ratio is not as impressive as the 6 : 1 ratio which has characterised lung cancer as predominantly a disease of males. Consequently, it would seem to be an overstatement to say, as did Impellitier (1957), that " when a male patient is found to have an adenocarcinoma of the lung, one had better search elsewhere for a primary tumor ".

Of the 284 women, 167 had adenocarcinomatous growths, while 117 showed squamous-celled growths, the former predominating in the ratio 1·43 : 1. Thus, as Attia (1951) and Murray (1957) both stressed, the adenocarcinoma is the cell type found in excess in women, although Stalker's (1957) figures showed no such excess.

We may also look at the cell types from another angle. Of the 1299 cases of squamous cell carcinoma in the whole series, 1182 occurred in men and 117 in women—a ratio of 11·1 : 1. In contrast, of the 701 cases of adenocarcinoma, men (534 cases) outnumbered women (167 cases) in the ratio of only 3·2 : 1. These figures reflect the present trend in lung cancer incidence (Ferrari and Kreyberg, 1960), namely, the increasing incidence of squamous cell carcinoma in men which is not paralleled by the adenocarcinoma.

### *Age*

The ages of the subjects were not available in 16 cases of squamous cell carcinoma and in 6 cases of adenocarcinoma. The remaining 1978 cases have been analysed to show the numbers of patients dying with either type of tumour in each of six age groups (Table II). Both types of tumour each contributed one case in the 3rd and 10th decades ; these individual cases have been combined with the cases occurring in the next age group for ease of calculation.

TABLE II.—*The Distribution of the Cell Types according to Age*

| Cell type | | Age groups in decades: 3, 4 | 5 | 6 | 7 | 8 | 9, 10 | Totals |
|---|---|---|---|---|---|---|---|---|
| Squamous cell carcinoma | (i) Number | 26 | 172 | 422 | 478 | 170 | 15 | 1283 |
| | (ii) Percentage | 2·0 | 13·4 | 32·9 | 37·3 | 13·3 | 1·2 | |
| Adenocarcinoma | (i) Number | 26 | 102 | 235 | 218 | 99 | 15 | 695 |
| | (ii) Percentage | 3·7 | 14·7 | 33·8 | 31·4 | 14·2 | 2·2 | |
| Contributions to $\chi^2$ | | 5·04 | 0·52 | 0·11 | 4·44 | 0·33 | 2·91 | 13·35 |

Table II shows that the percentage incidence of the adenocarcinoma is higher than that of the squamous-celled tumour, not only in the younger but also in the older age groups. Indeed, it is only in the 7th decade that the squamous cell carcinoma shows a higher incidence. We may test the hypothesis that for each decade the respective totals of the squamous-celled tumours and the adenocarcinomas should bear to one another the same ratio as their overall totals, namely, 1283 : 695. As Table II shows, the $\chi^2$ value, 13·35, is statistically significant, since $p < 0{\cdot}05$, for 5 degrees of freedom. This suggests that the differences between the cell types have not been caused by chance. The greatest departures from expectation occur in the 3rd and 4th, 7th and 9th and 10th decades. These results do not support the opinion of Judd (1947) that squamous-celled tumours occur more frequently in the older age groups than the adenocarcinomas. Galluzi and Payne (1955) did not find an excess of squamous cell carcinomas in older persons.

*Extrathoracic metastases*

The facility with which the two tumour types spread to extrathoracic sites was compared by reference to the invasion of seven single or paired organs, namely, liver, adrenal, brain, kidney, pancreas, thyroid and spleen (Table III). None of the tumours spread to all seven organs in any one individual. In over half of the squamous-celled tumours (53·2 per cent) no metastases had been demonstrated in the seven target organs ; the corresponding percentage for the adenocarcinomas is 28·8.

TABLE III.—*Influence of Cell Type on Number of Organs Showing Metastases*

| Cell type | | Number of invaded organs in each subject: 0 | 1 | 2 | 3 | 4 | 5 | 6 | Totals |
|---|---|---|---|---|---|---|---|---|---|
| Squamous cell carcinoma | (i) Number | 691 | 295 | 187 | 91 | 23 | 10 | 2 | 1299 |
| | (ii) Percentage | 53·2 | 22·7 | 14·4 | 7·0 | 1·8 | 0·8 | 0·2 | |
| Adenocarcinoma | (i) Number | 202 | 215 | 142 | 88 | 36 | 15 | 3 | 701 |
| | (ii) Percentage | 28·8 | 30·7 | 20·3 | 12·6 | 5·1 | 2·1 | 0·4 | |

As is true of the average case of lung cancer (Onuigbo, 1961*a*), both squamous-celled and adenocarcinomatous tumours tend to spread to a limited number of organs. The adenocarcinomas show, on the whole, a consistently higher rate of metastasis. The difference in behaviour may also be appreciated by enumerating all the organs invaded by each tumour type : the 1299 squamous-celled tumours invaded 1096 organs and the 701 adenocarcinomas attacked 1000 organs, the averages being respectively 0·84 and 1·43.

Koletsky (1938), who studied 100 cases of lung cancer, recorded the presence of extrathoracic metastases in 35 per cent of squamous cell carcinoma as against 86 per cent of the adenocarcinomas ; the corresponding percentages obtained by Auerbach (1949) in his series of 50 cases were 17 and 75. Hence, although the results differ, there is general agreement that adenocarcinomas show a greater tendency to spread outside the thorax than the squamous cell carcinomas.

*Organ selectivity*

Next, let us consider whether the two cell types display any evidence of organ selectivity in their metastasis. In other words, do the parenchymas of different organs offer each tumour type appreciably different soils for growth? Two lines of inquiry may be employed :

(1) We may obtain the percentage incidence of metastasis in each target organ, basing the calculation on the total number of cases of each cell type (Table IV). We find that this method of inquiry creates the impression that the adeno-

TABLE IV.—*Influence of Cell Type on Frequency of Metastasis of 7 Target Organs*

| Cell type | | Liver | Adrenal | Kidney | Brain | Pancreas | Spleen | Thyroid | Total cases |
|---|---|---|---|---|---|---|---|---|---|
| Squamous cell | (i) Number | 323 | 299 | 217 | 160 | 42 | 34 | 21 | 1299 |
| carcinoma | (ii) Percentage | 24·9 | 23·0 | 16·7 | 12·2 | 3·2 | 2·6 | 1·6 | |
| Adenocarcinoma | (i) Number | 280 | 289 | 149 | 171 | 62 | 29 | 20 | 701 |
| | (ii) Percentage | 39·9 | 41·2 | 21·3 | 24·4 | 8·8 | 4·1 | 2·9 | |

carcinoma shows in each organ a greater metastatic proclivity than the squamous cell carcinoma. This raises difficulties. For example, are we to conclude, as did Strauss and Weller (1957), that the adenocarcinoma shows about twice the propensity of the squamous cell carcinoma to spread to the adrenal?

(2) A valid concept of the comparative tendency of metastasis must take into account the extent to which each cell type failed to spread. We should assess only cases in which metastasis has actually occurred, i.e., those cases in which, as it were, the selection of organs has been made by the tumour cells. Table V shows the percentage incidence of invasion of the target organs calculated

TABLE V.—*Influence of Cell Type on Frequency of Metastasis of 7 Target Organs*

| Cell type | | Liver | Adrenal | Kidney | Brain | Pancreas | Spleen | Thyroid | Total organs |
|---|---|---|---|---|---|---|---|---|---|
| Squamous cell | (i) Number | 323 | 299 | 217 | 160 | 42 | 34 | 21 | 1096 |
| carcinoma | (ii) Percentage | 29·5 | 27·3 | 19·8 | 14·6 | 3·8 | 3·1 | 1·9 | |
| Adenocarcinoma | (i) Number | 280 | 289 | 149 | 171 | 62 | 29 | 20 | 1000 |
| | (ii) Percentage | 28·0 | 28·9 | 14·9 | 17·1 | 6·2 | 2·9 | 2·0 | |

on the total number of organs invaded by each cell type. We see that there is, in fact, close agreement between the two cell types with regard to the invasion of four organs, namely, liver, adrenal, thyroid and spleen, whereas the brain, kidney and pancreas show some divergence. Is the divergence statistically significant? On the hypothesis that for each organ the respective totals of the squamous cell carcinomas and the adenocarcinomas should bear to one another the same ratio as the overall totals, namely, 1096 : 1000, we find that the $\chi^2$

value, 16·14, is statistically significant ($p < 0{\cdot}02$, for 6 degrees of freedom). This indicates that there are differences in the organ selectivity of the two cell types. The organs showing the greatest departures from the 1096 : 1000 ratio are the kidney, pancreas and, to a lesser extent, brain.

Spencer (1962) noted that squamous-celled tumours seem to spread more often to the kidney than the other varieties of lung cancer. Galluzi and Payne (1955) did not find this relationship, nor did their figures show preponderance of adenocarcinomatous metastases in the pancreas ; they found that there was agreement between the different cell types in respect of the liver, adrenal, spleen and thyroid. Samson (1935) concluded that there was a strong tendency for adenocarcinomas not to spread to the spleen and pancreas. Tinney (1944) and Saphir (1958) affirmed that the histology was not a factor in the incidence of intracranial metastasis. In the opinion of Farber (1954), however, squamous cell carcinoma probably shows a predilection to spread to the brain, while Biggart (1949) indicted the adenocarcinoma. It seems to me that the present data accord with the view that the kidney provides a suitable soil for the growth of squamous cell carcinoma, and the pancreas and brain for the adenocarcinoma.

*Lateralisation of metastases*

In previous published analyses of metastases in the adrenal, liver, kidney and brain (Onuigbo, 1957*a*, 1957*b*, 1958*a*, 1958*b*), I suggested that in lung cancer these organs tend to be invaded characteristically—the deposits are more often wholly ipsilateral or larger ipsilaterally than contralaterally. It was postulated that such trends provide an overt evidence of the importance of lymphogenous metastasis to the viscera. It is of interest, therefore, to compare the behaviour of squamous cell carcinoma and adenocarcinoma in this respect.

TABLE VI.—*Influence of Cell Type on Lateralisation of Metastases*

| Cell type | | Liver | Adrenal | Kidney | Brain |
|---|---|---|---|---|---|
| Squamous cell carcinoma | (i) Ipsilateral | 48 | 120 | 70 | 70 |
| | (ii) Contralateral | 36 | 91 | 48 | 47 |
| | $\chi^2$ Value | 1·71 | 3·99 | 4·10 | 4·52 |
| Adenocarcinoma | (i) Ipsilateral | 30 | 93 | 39 | 73 |
| | (ii) Contralateral | 21 | 75 | 33 | 56 |
| | $\chi^2$ Value | 1·59 | 1·93 | 0·50 | 2·24 |

Table VI demonstrates that, with regard to the adenocarcinoma, the $\chi^2$ test does not reveal significant departures from expectations on the hypothesis that for each organ ipsilateral : contralateral : : 1 : 1, since we must have $\chi^2 > 3{\cdot}84$, for $p < 0{\cdot}05$ and one degree of freedom. In contrast, in respect of the squamous cell carcinoma, there are significant $\chi^2$ values for the adrenal, kidney and brain, but not for the liver.

How are these results to be interpreted? As with all tests of significance—see Hill (1958)—our conclusions must turn on probabilities. Since the study of lymph node metastases suggests that large statistical differences in lateralisation of visceral metastases should not be expected (Onuigbo, 1961*c*), I think that a valid generalisation from the above data is that squamous cell carcinomas do show the ipsilateral metastatic trend much more than the adenocarcinomas. Furthermore, the hypothesis may be put forward that, if the ipsilateral trend is to some extent indicative of lymphogenous spread, then the squamous cell carcinoma is the cell type that spreads more easily by this route, while the adenocarcinoma is perhaps the type that is more able to colonise organs haphazardly by way of the blood stream. This hypothesis is advanced for concerted consideration because, as I have suggested elsewhere (Onuigbo, 1961*b*, 1962), new prospects may open in cancer research if comparable attention is paid to not only the haematogenous but also the lymphogenous theory of metastasis.

### SUMMARY

A salient feature of the published statistical analyses of lung cancer is the conflict of opinion on many problems. One area of conflict is the influence of the cell type on the pathology of this tumour. Accordingly, 2000 cases of which 1299 were squamous cell carcinomas and 701 were adenocarcinomas were collected during a country-wide survey of the necropsy records of 27 medical schools and 7 associated hospitals. A detailed comparison of the two cell types was carried out with special reference to sex, age, extrathoracic metastases, organ selectivity and laterality of the metastases of paired organs.

There were 1716 men and 284 women, a ratio of 6·04 : 1. Among the men, 1182 exhibited squamous-celled tumours and 534 showed adenocarcinomatous tumours ; the corresponding figures for women were 117 and 167. Attention is drawn, therefore, to the error of regarding adenocarcinomas as a rare tumour type in males with lung cancer.

When 1978 cases with known ages were classified by decades into six age groups, it was found that the percentage incidence of the adenocarciomas was higher than that of the squamous cell carcinomas not only in the younger but also in the older age groups, except in the 7th decade. The differences between the two cell types were statistically significant.

53·2 per cent of the squamous cell carcinomas did not spread to any of seven listed organs, the corresponding percentage for the adenocarcinomas being only 28·8. In other respects the latter tumours showed a consistently higher rate of extrathoracic metastasis.

There was a close agreement between the two cell types in respect of their selection of the liver, adrenal, thyroid, and spleen as sites for metastasis. The kidney appeared to offer more fertile soil to the squamous-celled tumours, and the pancreas and brain to the adenocarcinomas.

Squamous cell carcinomas showed a statistically significant tendency to spread to the ipsilateral adrenal, kidney and brain in contrast to the adenocarcinomas. It is suggested, therefore, that if ipsilateral metastasis were an overt evidence of lymphogenous spread then the probability was that squamous cell carcinomas spread in this way as opposed to the adenocarcinomas which are perhaps more able to colonise organs haphazardly by way of the blood stream. It is thought that there is need to explore such leads which may open new prospects in cancer research.

For access to the necropsy records I am indebted to Professor D. F. Cappell, my chief, and to Professors J. W. S. Blacklock, Sir Roy Cameron, A. C. P. Campbell, D. H. Collins, T. Crawford, R. C. Curran, J. B. Duguid, J. Gough, C. V.

Harrison, T. F. Hewer, K. R. Hill, A. C. Lendrum, C. E. Lumsden, H. A. Magnus, J. W. Orr, G. L. Montgomery, D. M. Pryce, R. J. V. Pulvertaft, D. S. Russell, R. W. Scarff, H. L. Sheehan, T. Symington, W. St. C. Symmers, G. P. Wright and J. S. Young, and to Doctors A. M. Barrett, W. B. Davies, A. Dick, J. C. Dick, J. M. Drennan, W. Goldie and A. H. T. Robb-Smith. Dr. R. A. Robb of the Statistics Department of the University of Glasgow gave me much useful criticism and advice.

### REFERENCES

ATTIA, O. M.—(1951) Ph.D. Thesis, University of Edinburgh.
AUERBACH, O.—(1949) *N.Y. St. J. Med.*, **49**, 900.
BERKSON, N.—(1953) *Canad. med. Ass. J.*, **69**, 406.
BIGGART, J. H.—(1949) 'Pathology of the Nervous System'. Edinburgh (Livingstone), p. 321.
BUDINGER, J. M.—(1958) *Cancer*, **11**, 106.
FARBER, S. M.—(1954) 'Lung Cancer'. Springfield (Thomas), p. 40 ff.
FERRARI, E. AND KREYBERG, L.—(1960) *Brit. J. Cancer*, **14**, 609.
GALLUZI, S. AND PAYNE, P. M.—(1955) *Ibid.*, **9**, 511.
HILL, A. B.—(1958) 'Principles of Medical Statistics'. London (*The Lancet*), p. 125.
IMPELLITIER, C. J.—(1957) *N.Y. St. J. Med.*, **57**, 2406.
JUDD, A. R.—(1947) 'Diseases of the Chest'. Philadelphia (Davis), p. 423.
KOLETSKY, S.—(1938) *Arch. intern. Med.*, **62**, 636.
MURRAY, F.—(1957) In 'Bronchopulmonary Diseases'. (Edited by Naclerio), London (Cassell) p. 695.
NOHL, H. C.—(1962) 'The Spread of Carcinoma of the Bronchus'. London (Lloyd-Luke), pp. 19–21.
ONUIGBO, W. I. B.—(1957*a*) *Brit. J. Cancer*, **11**, 175.—(1957*b*) *W. Afr. med. J.*, **6**, 175.—(1958*a*) *Cancer*, **11**, 737.—(1958*b*) *Brit. J. Tuberc.*, **52**, 141.—(1961*a*) *Tubercle*, **42**, 248.—(1961*b*) *Cancer Res.*, **21**, 1077.—(1961*c*) Ph.D. Thesis, University of London.—(1962) *Z. Krebsforsch.*, **65**, 30.
SAMSON, P. C.—(1935) *Amer. J. Cancer*, **23**, 754.
SAPHIR, O.—(1958) 'A Text on Systematic Pathology'. New York (Grune and Stratton), vol. 1, p. 347.
SPENCER, H.—(1962) 'Pathology of the Lung'. Oxford (Pergamon), p. 672.
STALKER, R.—(1957) M.D. Thesis, University of Edinburgh.
STRAUSS, B. AND WELLER, C.—(1957) *Arch. Path.*, **63**, 602.
TINNEY, W. S.—(1944) *Arch. Otolaryng., Chicago*, **39**, 243.


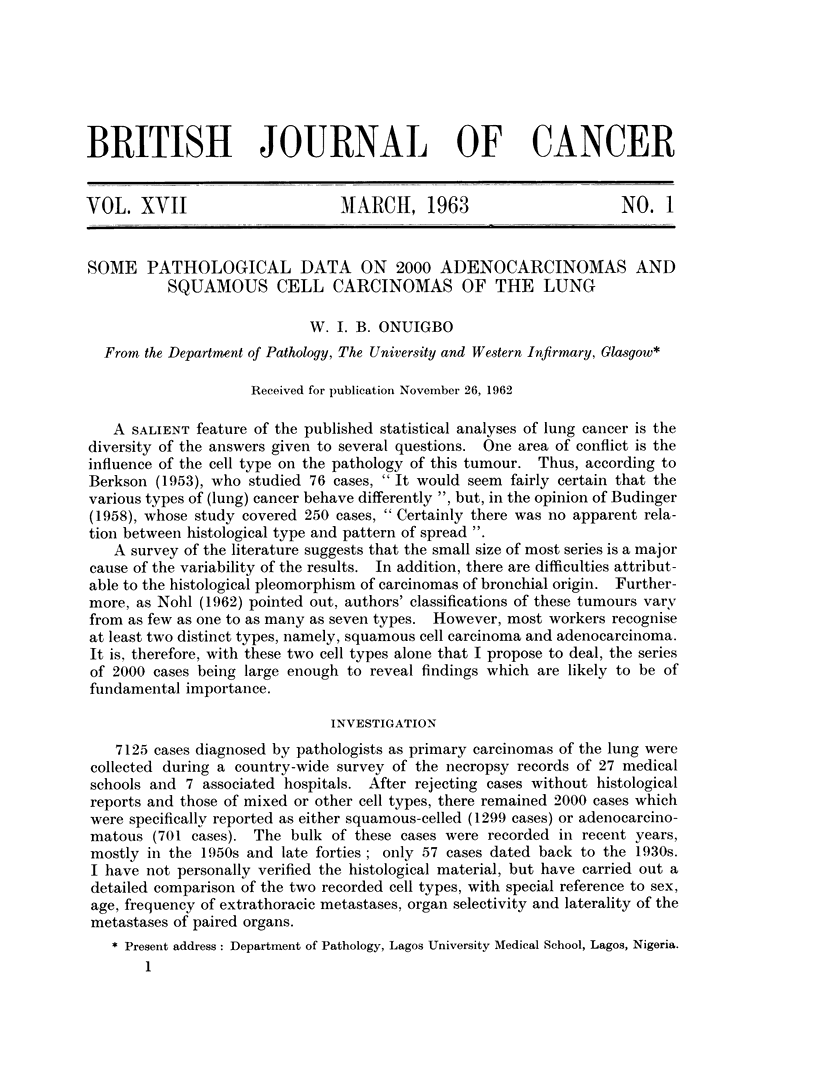

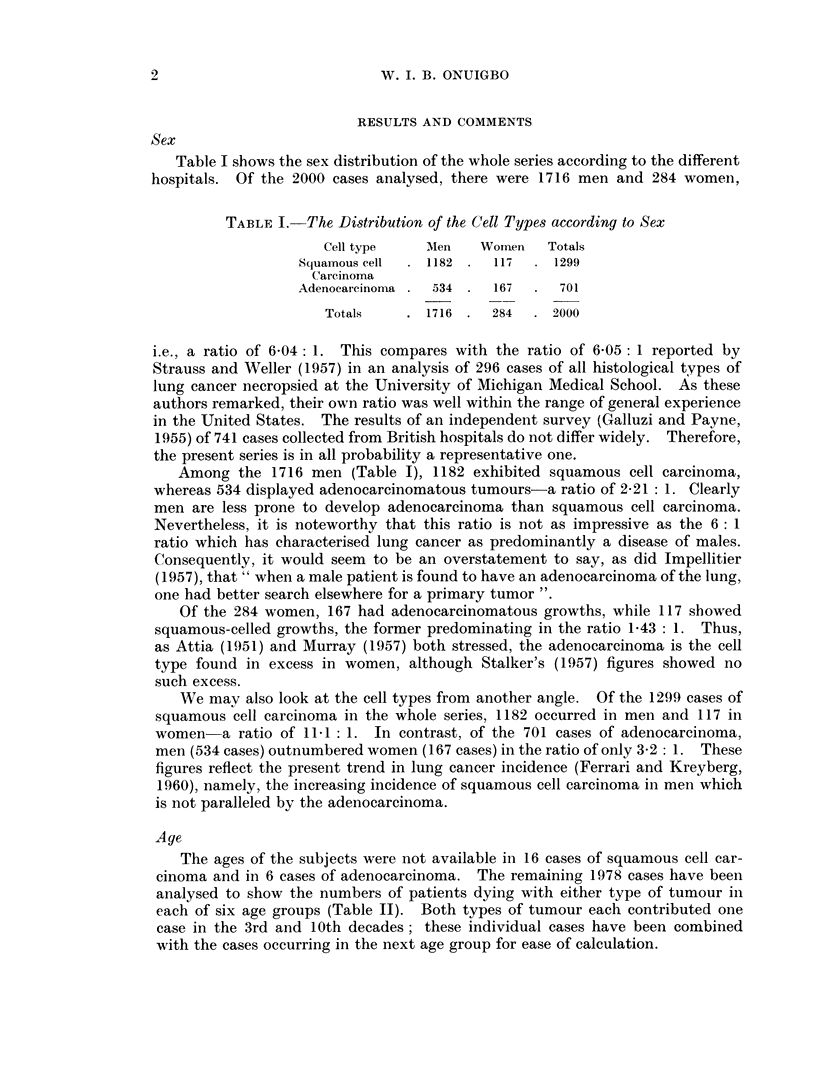

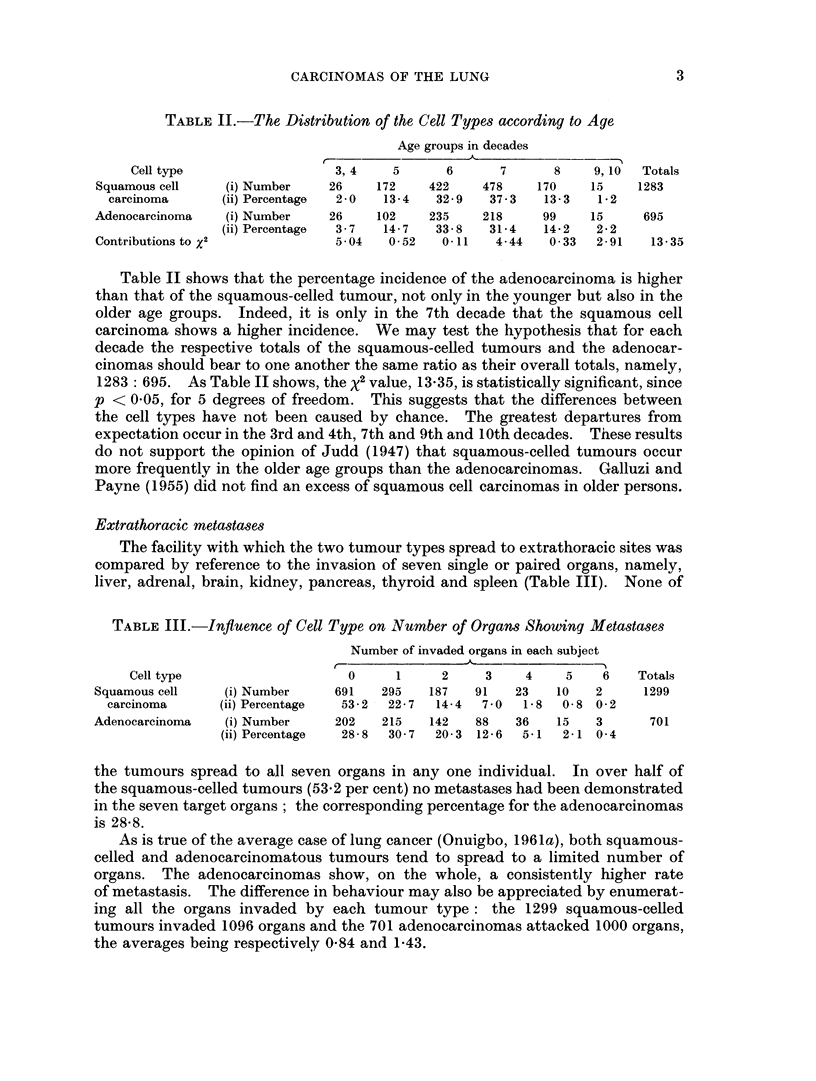

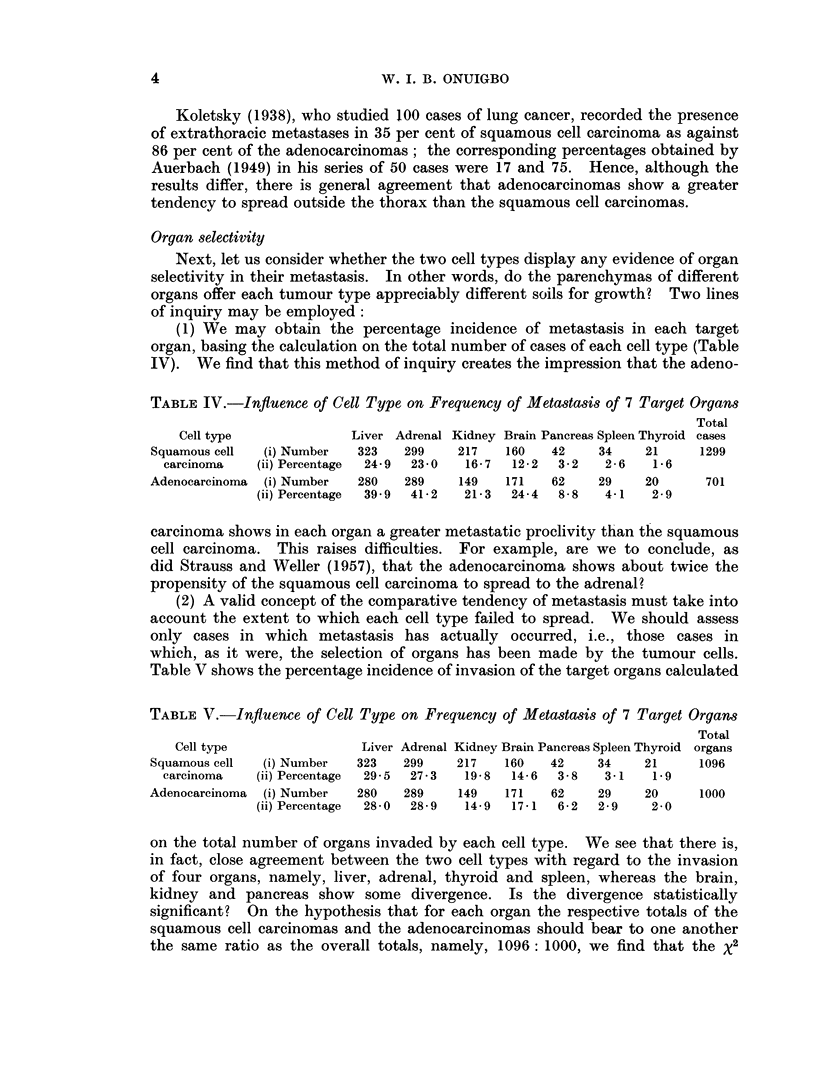

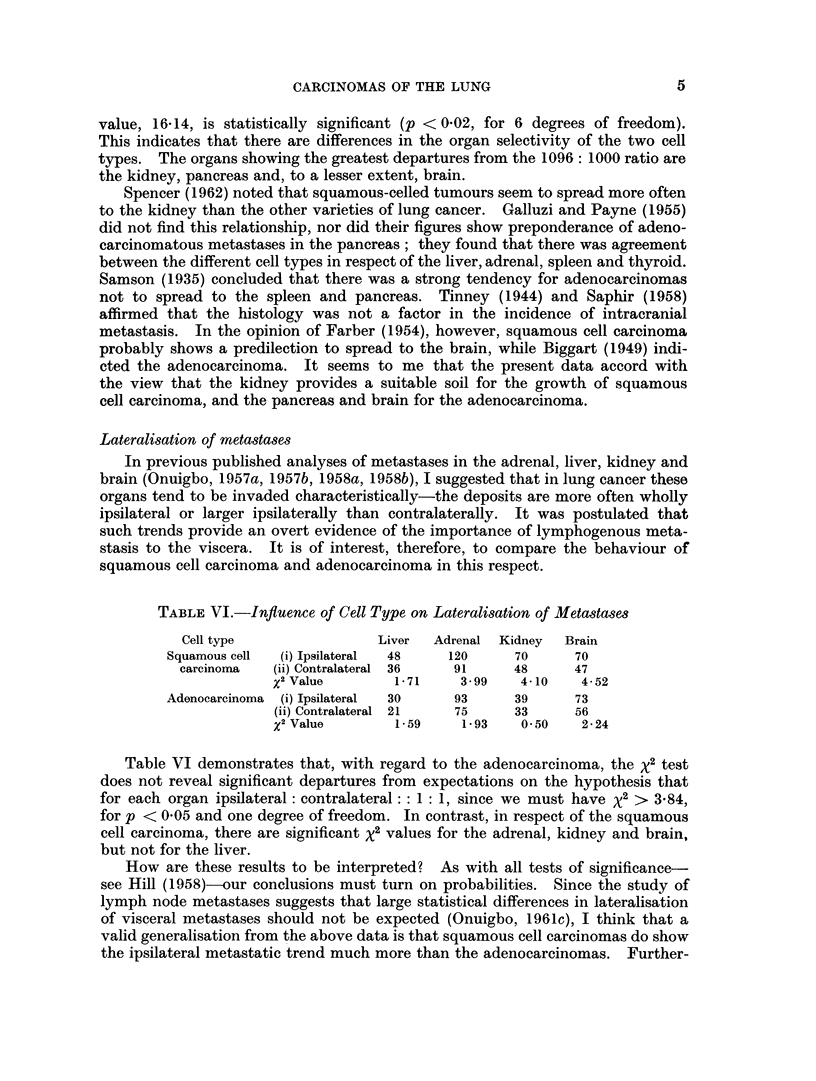

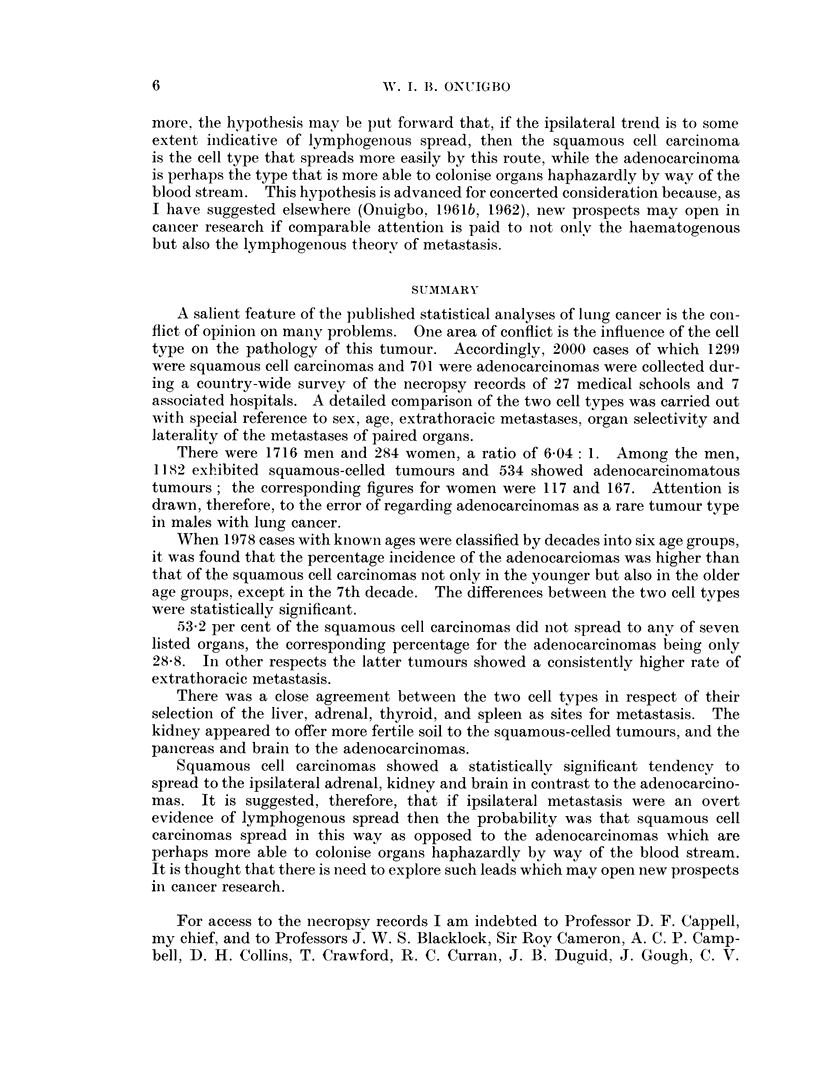

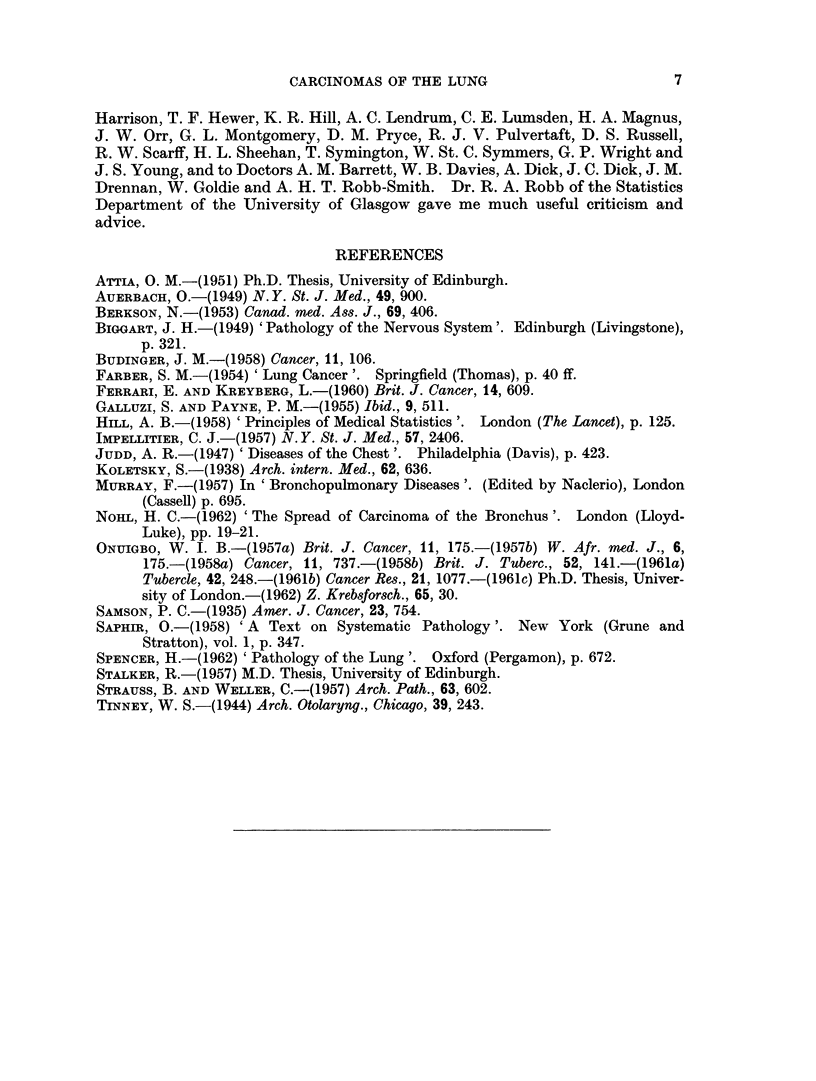


## References

[OCR_00380] BERKSON N. (1953). Bronchogenic carcinoma.. Can Med Assoc J.

[OCR_00384] BUDINGER J. M. (1958). Untreated bronchogenic carcinoma; a clinicopathological study of 250 autopsied cases.. Cancer.

[OCR_00388] FERRARI E., KREYBERG L. (1960). Histological types in a lung cancer material in Venice.. Br J Cancer.

[OCR_00417] STRAUSS B., WELLER C. V. (1957). Bronchogenic carcinoma; a statistical analysis of two hundred ninety-six cases with necropsy as to relationships between cell types and age, sex, and metastasis.. AMA Arch Pathol.

